# Identification of serum biomarkers for cystic echinococcosis in sheep through untargeted metabolomic analysis using LC–MS/MS technology

**DOI:** 10.1186/s13071-024-06599-6

**Published:** 2024-12-30

**Authors:** Xiao-Xia Wu, Wan-Li Ban, Li-Jiang Wu, Wen-Jing Qi, Mehdi Borhani, Xiao-Ying He, Xiao-Lei Liu, Ming-Yuan Liu, Jing Ding

**Affiliations:** 1 State Key Laboratory for Diagnosis and Treatment of Severe Zoonotic Infectious Diseases, Key Laboratory for Zoonosis Research of the Ministry of Education, Institute of Zoonosis and College of Veterinary Medicine, Jilin University, Changchun, 130062 China; 2 National Animal Echinococcosis Reference Laboratory, Veterinary Research Institute of Xinjiang Academy of Animal Husbandry, Urumqi, 830010 China; 3 State Key Laboratory of Pathogenesis, Prevention and Treatment of High Incidence Diseases in Central Asia First Affiliated Hospital of Xinjiang Medical University, Urumqi, 830054 China

**Keywords:** *Echinococcus granulosus*, Serum, Non-targeted metabolomics, LC–MS/MS, Biomarkers

## Abstract

**Background:**

Echinococcosis is a zoonotic disease caused by an *Echinococcus* tapeworm infection. While diagnostic methods for humans often rely on ultrasound imaging and immunodiagnostic techniques, diagnosis in intermediate hosts typically has no widely used diagnostic markers, hampering disease control efforts.

**Methods:**

The differences in serum metabolites of sheep infected with *Echinococcus granulosus* and a control group were analyzed using ultrahigh-performance liquid chromatography (UHPLC) separation with tandem mass spectrometry (MS/MS) detection. This provided a basis for the early diagnosis and pathogenetic study of cystic echinococcosis (CE) in intermediate hosts at the metabolomics level. Orthogonal projections to latent structures–discriminant analysis (OPLS-DA) were used to analyze different metabolites in the serum of the two groups. The differentially abundant metabolites were entered into the MetaboAnalyst 5.0 online analysis website for processing, and the top-15-ranked metabolic pathways were set to produce bubble plots and differential abundance score plots, with a significant difference of *P* < 0.05 and a false discovery rate (FDR) < 0.1 as the screening conditions.

**Results:**

Data analyses of serum samples from both groups identified a total of 1905 significantly different metabolites, where 841 metabolites were upregulated and 1064 metabolites were downregulated. Twelve metabolites were significantly upregulated and 21 metabolites were significantly downregulated in the experimental group. Then, the 1,7-dihydroxyxanthone, 2-methylbutyrylglycine, 3,3-dimethylglutaric acid, 5,12-dihydroxy-6,8,10,14,17-eicosapentaenoic acid, 9-hydroperoxy-10E,12Z,15Z-octadecatrienoic acid, and trimethylamine *N*-oxide 6 metabolites were selected as diagnostically valuable candidate biomarkers (area under the curve [AUC] > 0.7). These differential metabolites are involved in various metabolic pathways, including amino acid metabolites (arginine, l-isoleucine, l-valine) and fatty acid metabolism (fenugreek, arachidonic acid, linolenic acid). Compared with the control group, sheep in the CE group had increased serum levels of fenugreek acid, while all other metabolites such as glycine showed significantly reduced serum levels (*P* < 0.01).

**Conclusions:**

Through non-targeted metabolomic analysis of the serum of CE-infected sheep, differential metabolites closely related to amino acid metabolism and the fatty acid metabolism pathway were identified. These differentially abundant metabolites can serve as biomarkers for diagnosing CE infection in intermediate sheep hosts.

**Graphical abstract:**

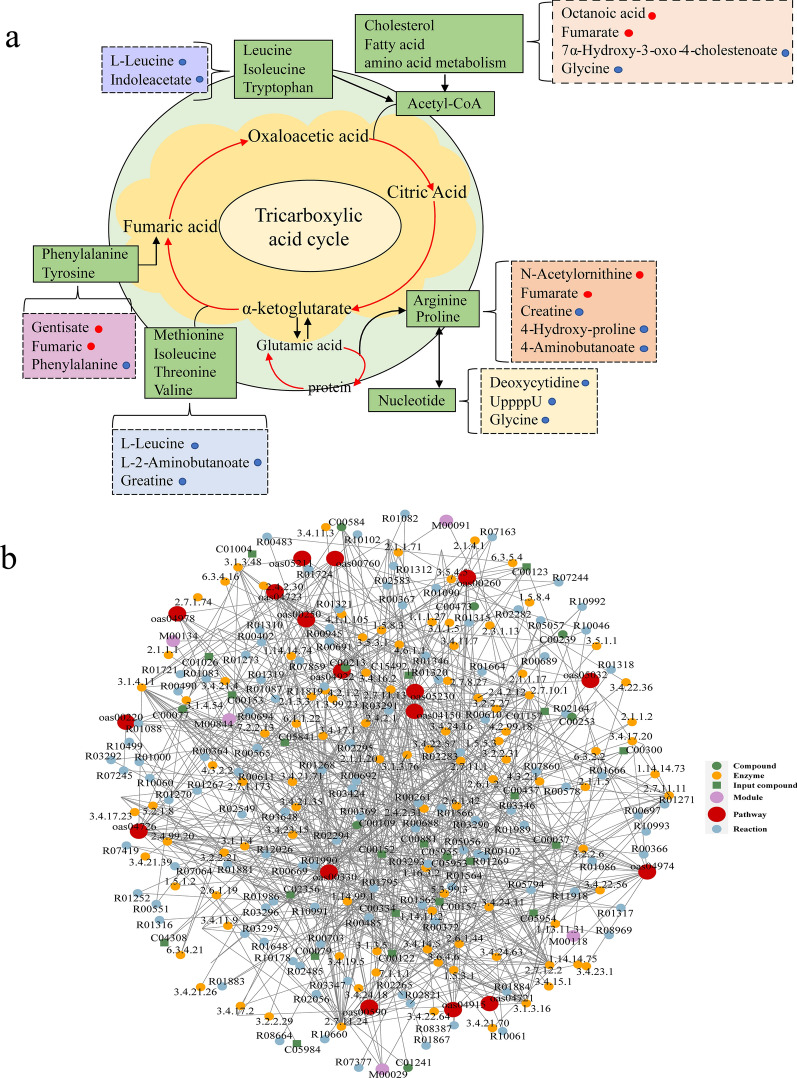

## Background

Hydatidosis, also known as cystic echinococcosis (CE), is a parasitic disease caused by infection with the larvae of tapeworms belonging to *Echinococcus granulosus*. Hydatid disease is a significant health concern, with two primary types of pathogens contributing to its prevalence: CE caused by *E. granulosus* sensu lato and alveolar echinococcosis attributed to *Echinococcus multilocularis*. *E. granulosus* specifically stands out as the predominant pathogen responsible for hydatid disease worldwide [1–4].

Cystic echinococcosis is widespread and prevalent in many regions globally, particularly affecting rural environments in Africa, the Middle East, Mediterranean Europe, Central Asia, South America, and western China, where humans and animals closely coexist [5]. According to epidemiological surveys conducted by the Chinese Center for Disease Control and Prevention at the end of 2022, 370 counties in China were endemic for CE, with 25,227 cases of echinococcosis recorded. Moreover, the detection rate of infected livestock stood at 0.88%, with 1038 cases identified out of 117,303 animals examined [6–8]. The World Health Organization reports that approximately one million individuals globally are infected with CE, leading to an estimated burden of 183,573 (ranging from 88,082 to 1,590,846) disability-adjusted life years attributed to this zoonotic infection [9]. The economic impact of the disease, inclusive of treatment expenses and losses in livestock, is estimated at US$3 billion annually. This disease incurs significant financial costs in low- and middle-income countries, amounting to ~ 0.01–0.04% of a nation’s gross domestic product [10].

Given these circumstances, diagnosing this disease is vital for epidemiological research, disease surveillance, and control efforts. In humans, ultrasound imaging and immunodiagnostic techniques are considered the most dependable methods for diagnosing echinococcosis. However, accurately detecting echinococcosis lesions during their inactive phases with sonography is challenging due to the complex nature of CE. In contrast to human research efforts, limited attention has been given to the advancement of immunodiagnostic techniques for detecting echinococcosis infections in host animals such as sheep and cattle. At present, the diagnosis of echinococcosis in intermediate hosts relies primarily on necropsy methods, with a notable absence in laboratory diagnostic methods that significantly influence the efficacy of national disease control strategies [11].

Therefore, the identification of dependable biomarkers related to CE in intermediate hosts is crucial to enable early detection and treatment. The liver plays a key role in multiple metabolic processes and, as the central site for *E. granulosus* infection, results in significant metabolic changes in the serum when damaged [12, 13]. Omics technologies, including genomics, proteomics, and metabolomics, play a pivotal role in elucidating these complex aspects, aiding in the identification of potential biomarkers and facilitating the development of effective drugs, precise disease diagnosis, and vaccine targets [14–17].

Here, liquid chromatography–mass spectrometry (LC–MS), a key metabolomic technique that holds unparalleled potential in unraveling the intricacies of the metabolome [18–20], is widely employed to analyze metabolites in the sera of patients infected with *Trichinella spiralis*, *Treponema pallidum*, and vesicular echinococcosis [21–25]. Untargeted metabolomics using LC–MS enables the measurement of numerous metabolic features from biological samples. Zhu et al. [26], in 2019, identified the metabolites consumed and released by *E. multilocularis* under anaerobic conditions and showed that glucose was the most consumed metabolite. Additionally, examining metabolic changes in the serum can directly indicate the severity of CE [27]. While some studies have highlighted the metabolic shifts in patients with this disease, a thorough understanding of metabolic variations in the liver is still lacking [28]. Unraveling these metabolic changes holds promise for illuminating the disease’s progression and uncovering novel biomarkers for clinical diagnosis [12]. Recent findings reveal significant differences in serum metabolic profiles in advanced stages of CE, yet detailed metabolic variations in early-stage serum remain ambiguous [24, 29].

Despite the progress made in scientific research, the quest for an effective and non-invasive biomarker for the early detection of CE in sheep continues. Here, metabolites and biochemical indices emerge as key indicators of disease responses, offering valuable clues about the underlying pathophysiological processes. Integrating these two components could unlock new scientific dimensions, paving the way for enhanced diagnostics and tailored interventions. Therefore, a comprehensive non-targeted metabolomic analysis of serum from sheep with CE could unveil crucial disease biomarkers. This study aimed to explore the metabolic alterations underlying the disease’s progression in sheep infected with CE to identify diagnostic biomarkers. The findings of this study will increase our understanding of CE at the metabolic level and provide reliable biomarkers for early diagnosis of the disease in clinical settings. Current diagnostic methods for CE in intermediate hosts lack effectiveness, creating challenges in disease control and management. The discovery of these biomarkers could lead to improved diagnostic protocols, influencing both animal health outcomes and the economic stability of regions where CE is endemic.

## Methods

### Serum sample collection, histology, and histomorphometry

Female sheep (2–4 months old) from Changchun, Jilin province, were provided by a local vendor. Sheep were maintained on forage grass and tap water. Following a period of adaptation, test sheep were artificially infected orally with 1000 *E. granulosus* eggs. After 180 days, animals were euthanized and positive serum was collected. Then, autopsies were performed and the livers and lungs were removed for examination of the cystic condition. Serum samples from a total of two groups of sheep were selected for the experiment, with six biological replicates in each group. There were six serum samples from diseased sheep, and another six serum samples from healthy sheep during the same period were selected as the healthy control group, for a total of 12 samples.

After necropsy, sheep hydatid cyst samples were collected, fixed in 4% paraformaldehyde, dehydrated using an ethanol gradient, and embedded in paraffin. Subsequently, continuous sections with a thickness of 4 μm were prepared and stained with hematoxylin and eosin (H&E) as well as Masson’s trichrome staining.

### Reagents and instruments

Class LC–MS-grade ammonium acetate, metabolite internal standards, and ammonium hydroxide solutions were procured from Sigma-Aldrich (Watertown, MA, USA). For metabolomic analysis, an ultrahigh-performance liquid chromatography (UHPLC) system (Vanquish) coupled with tandem high-resolution MS (Orbitrap Exploris 120, Thermo Fisher Scientific) was employed.

### Metabolite extraction

One hundred microliters of serum was mixed with 400 μl of extraction solution (MeOH:ACN, 1:1[v/v]) in a 1.5 ml centrifuge tube, vortexed for 30 s, sonicated for 10 min in 4 °C water bath, incubated for 1 h at −40 °C to precipitate proteins, and then centrifuged at 4 °C for 15 min at 12,000 r/min (relative centrifugal force [RCF] = 13,800×*g*, radius [R] = 8.6 cm). All the supernatant was transferred to a fresh glass vial for analysis. The quality control (QC) sample was prepared by mixing an equal aliquot of the supernatant of the samples. The analysis was performed based on liquid chromatography–tandem MS (LC–MS/MS). Finally, all samples to be measured were mixed to a volume equal to the QC samples, to evaluate the stability of the system throughout the experiment [30].

### LC–MS/MS analysis

For polar metabolites, LC–MS/MS analyses were performed using a UHPLC system (Vanquish, Thermo Fisher Scientific) with a Waters ACQUITY UPLC BEH Amide (2.1 mm × 50 mm, 1.7 μm) coupled to an Orbitrap Exploris 120 mass spectrometer (Thermo Fisher Scientific) [31]. The mobile phase consisted of 25 mmol/l ammonium acetate and 25 mmol/l ammonia hydroxide in water (pH = 9.75) (A) and acetonitrile (B). The autosampler temperature was 4 °C, and the injection volume was 2 μl. The gradient elution program was as follows: 0–2 min, 5% mobile phase B; 2–4 min, 30% mobile phase B; 4–8 min, 50% mobile phase B; 8–10 min, 80% mobile phase B; 10–15 min, 100% mobile phase B; and 15–16 min, 5% mobile phase B. The flow rate was 0.35 ml/min [30].

The Orbitrap Exploris 120 mass spectrometer was used for its ability to acquire MS/MS spectra in information-dependent acquisition (IDA) mode via acquisition software (Xcalibur, version: 4.4, Thermo). In this mode, the acquisition software continuously evaluates the full-scan MS spectrum. The MS conditions were as follows: an electrospray ionization (ESI) ion source was employed, and positive and negative ion scanning modes were used to collect sample quality spectrum signals. The ESI source conditions were set as follows: sheath gas flow rate, 50 Arb; auxiliary gas flow rate, 15 Arb; capillary temperature, 320 °C; full MS resolution, 60,000; MS/MS resolution, 15,000; collision energy, 20/30/40; and spray voltage, 3.8 kV (positive) or −3.4 kV (negative) [32].

### Metabolite identification and data analysis

The spectral data collected by LC–MS were converted into mzXML format using ProteoWizard software, and MS-DIAL software was used to identify and compare the detected peaks. Then a dataset consisting of the name, group, and peak height was obtained [32]. Multivariate statistical analysis was performed by unsupervised principal component analysis (PCA) to observe the overall distribution among samples and the stability of the whole analysis process, followed by orthogonal projections to latent structures–discriminant analysis (OPLS-DA) to analyze the results. The data were formatted using SIMCA software (v.16.0.2, Sartorius Stedim Data Analytics AB, Umea, Sweden) with logarithmic transformation plus centering, and then subjected to automated modeling analysis [33].

The results were analyzed by OPLS-DA. Finally, the data were subjected to total ion flow normalization, log transformation, and Pareto calibration. Furthermore, the data were found to follow a normal distribution and were then analyzed to identify any significant differences in metabolites between the model group and the CE group, with significance denoted by *P* < 0.05. Intergroup differentially abundant metabolites were identified and annotated using the Kyoto Encyclopedia of Genes and Genomes (KEGG) database, and clustering analysis and metabolic pathway enrichment were performed using the MetaboAnalyst 5.0 online website (http://www.metaboanalyst.ca/). MetaboAnalyst 5.0 was also used for cluster analysis, regulatory network analysis, and metabolic pathway enrichment. In addition, a receiver operating characteristic (ROC) curve was generated to evaluate the sensitivity and specificity of each metabolite. The area under the ROC curve (AUC) was calculated for the CE group and the control group, and a metabolite was indicated to have high diagnostic performance when the AUC was ≥ 0.8 [34].

### Statistical analysis

All the data were statistically analyzed using SPSS version 22.0 and GraphPad Prism 9.3.1 software. Diagnostic reference screening and performance assessment potential were determined by evaluating variable importance for the projection (VIP) > 1.0 obtained from the OPLS-DA model. The differences between the CE and control groups were compared using Student’s *t*-test for continuous variables, coefficient of variation (CV)-analysis of variance (ANOVA) for continuous variables, and the Chi-square test for categorical variables. Here, *P* < 0.05 was considered statistically significant [32, 35].

## Results

### Pathological morphology analysis of echinococcosis intermediate hosts

**Fig. 1:**
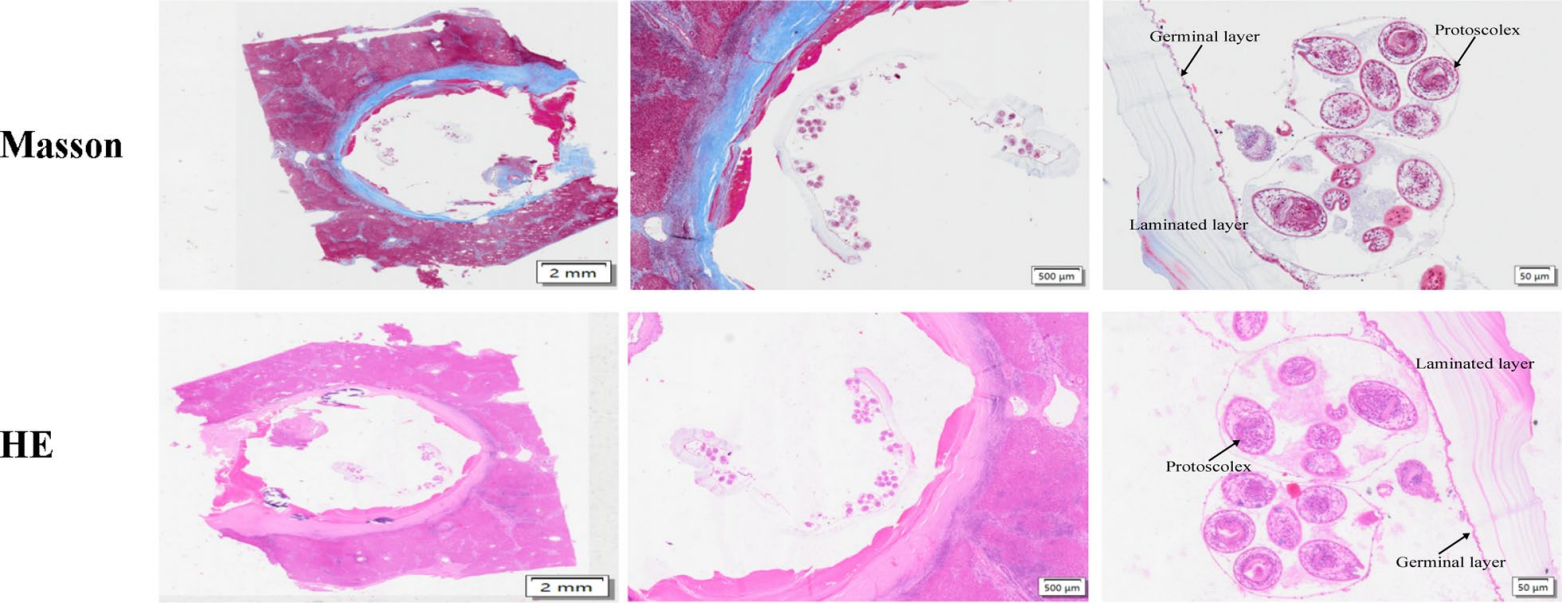
Pathological sections of hepatic cystic echinococcosis. Histopathological appearance: Hydatid cyst with protoscolex within the liver, demonstrated by Masson staining. Histopathological appearance: Hydatid cyst with protoscolex within the liver, visualized using hematoxylin–eosin (H&E) staining

The cyst samples were examined histopathologically, and the staining results, including H&E and Masson’s, were found to be consistent with the autopsy findings (Fig. 1). Notably, there was a well-defined fibrous capsule and inflammatory lesions present in the liver tissue. The capsule wall was smooth and clear, with the germinal layer attached to the stratum corneum situated in the innermost part of the capsule cavity. The germinal layer comprised round nucleated cells and microscopic material at its base, possessing distinct embryonic tissue properties. This layer gave rise to a highly reproductive germinal capsule and protoscolex. The protoscolex exhibited invaginated structures, maintained their normal morphology, showed no significant changes, and appeared to be normally viable.

### PCA and OPLS-DA

**Fig. 2:**
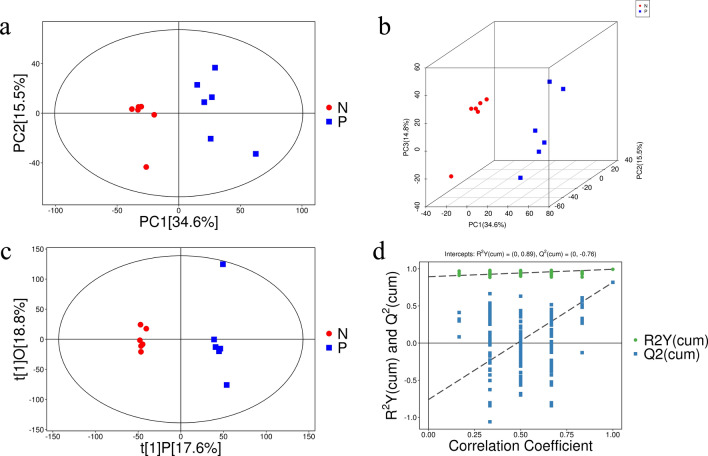
PCA diagram of sheep and OPLS-DA model 200 response ranking test. **a** PCA score plots of CE group and control group. **b** Three-dimensional (3D) PCA score plots of CE group and control group. The blue scatter points represent the CE model group, and the red represent the healthy control sample. The horizontal axis PC1 and the vertical axis PC2 represent the first- and second-ranked principal component scores, respectively. **c** Score scatter plot of OPLS-DA model for CE group and control group. The horizontal coordinate t[1]P represents the predicted principal component score of the first principal component, showing differences between sample groups; the ordinate t[1]O represents the orthogonal principal component score, showing differences within sample groups. **d** The OPLS-DA permutation plot of the serum for sheep infected with CE and control groups in intercept mode with R2Y(cum) = (0, 0.89), Q2(cum) = (0, −0.76), and correlation coefficient (0.25–0.9)

PCA was conducted to assess the stability of the analytical system and observe the trends in sample distribution under stable and reproducible conditions. The data were formatted using SIMCA software with log transformation plus centering, and then subjected to automated modeling analysis [36]. According to the scatter plot model of the PCA scores (Fig. 2a, b), the samples showed good aggregation in both the positive and negative ion modes, indicating that the analysis was stable and reliable. All samples fell within the 95% confidence intervals as depicted by Hotelling’s *t*-squared ellipse. Additionally, the test system demonstrated good reproducibility during batch analysis, satisfying the batch analysis requirements for metabolomics research.

To emphasize the differences between the two groups, serum metabolite prediction principal components were analyzed through OPLS-DA modeling. The findings revealed significant differences in the serum profiles between the CE group and the healthy group of sheep, as evidenced in the OPLS-DA score plot. The metabolic profiles could be clearly differentiated, the separation was good, and all the samples were uniformly distributed within the 95% confidence interval, with an R2X of 0.364, an R2Y of 0.993, and a cumulative prediction rate of 0.818, indicating that the OPLS-DA model was robust with good prediction ability (Fig. 2c). The accuracy of the model was tested by sevenfold cross-validation and 200-fold response ordering tests, and the regression line intercept of the constructed model together with the Y-axis cumulative explanatory rate (R2) of the real model was reported as 0.89. The regression line intercept of the constructed model together with the Y-axis average prediction rate (Q2) of the real model was −0.76 (Fig. 2d), indicating that the OPLS-DA model was not overfitted and had good predictive ability and stability.

### Screening and identification of biomarkers

Based on the serum differential metabolic profiles, 1905 differentially abundant metabolites were screened in the sera of the two groups, of which 841 metabolites were upregulated and 1064 metabolites were downregulated, using the screening criteria of *P* < 0.05 and variable significance projections > 1. Among these, 12 metabolites were significantly upregulated and 21 metabolites were significantly downregulated. The significantly different metabolites (*P* < 0.01 and VIP > 1) identified in the sera of the CE and control groups are shown in Table 1.

**Table 1:** Potential serum biomarkers for the diagnosis of the control and CE groups

ID	Metabolite	Control group/CE serum group			
		VIP	*P*-value	Log_2_ fold change	Up/down
226	Acetylcarnitine	2.01	< 0.01	1.12	↑
454	1,7-Dihydroxyxanthone	2.31	< 0.01	3.81	↑
792	*N*-(6-Amino-Bu-1,2,4-dioxo-THP)-*N*-MeBzam	2.38	< 0.01	10.11	↑
385	Prostaglandin B2	2.24	< 0.01	2.53	↑
425	Prostaglandin A2	2.24	< 0.01	2.53	↑
615	Spiro[3.3]Hpt-2,2-dicarboxylic acid	2.32	< 0.01	5.19	↑
965	beta-Damascone	2.17	< 0.01	3.62	↑
20	Trimethylamine *N*-oxide	2.28	0.01	4.59	↑
1087	5-Hydroxyflavone	2.31	< 0.01	7.56	↑
29	3,3-Dimethylglutaric acid	2.13	< 0.01	1.28	↑
427	2-Methylbutyrylglycine	2.08	< 0.01	1.29	↑
1464	dUMP	1.69	< 0.01	1.10	↑
1197	*O*-Desmethyl gefitinib	2.08	< 0.01	−3.45	↓
703	Tegaserod	2.05	< 0.01	−5.37	↓
766	Lactapiperanol_D	2.02	< 0.01	−2.58	↓
1001	(23S)-23,25-diOH-24-oxoVD3-23-beta-Glu	2.04	0.01	−3.04	↓
824	all-*trans*-13,14-Dihydroretinol	2.20	< 0.01	−7.50	↓
1336	14,16,17,20-OH-1,26-oxo-22,26-epoxyErg-5,24-dien-3-Glu	2.29	< 0.01	−6.37	↓
545	6,15-diketo-13,14-dihydro-PGF1α	2.28	< 0.01	−6.35	↓
1008	8,9-diOH-5Z,11Z,14Z-ETA	1.86	< 0.01	−3.89	↓
551	Trp-Lys	2.23	< 0.01	−4.91	↓
620	1α-Me-5α-andro-3α,17β-diol-Glu	2.17	< 0.01	−5.84	↓
1074	4E,6E-8-Glu-2,7-Me2-ODA	1.86	0.01	−4.38	↓
830	Lys-Arg	2.14	< 0.01	−2.78	↓
748	9-OOH-10E,12Z,15Z-octadecatrienoic acid	2.11	< 0.01	−1.32	↓
506	13,14-Dihydro prostaglandin F1 alpha	2.02	< 0.01	−2.53	↓
421	13,14-diH-15-keto-t-PGF1β	2.26	< 0.01	−2.56	↓
1311	Germanicol cinnamate	2.29	< 0.01	−3.90	↓
484	6b-Hydroxymethandienone	2.24	< 0.01	−6.46	↓
744	5,12-diOH-6,8,10,14,17-eicosapentaenoic_acid	2.19	< 0.01	−7.23	↓
968	Pen-Ind-3-CO-NH-Bn	2.36	< 0.01	−9.22	↓
706	15S-OOH-11Z,13E-eicosadienoic acid	2.01	< 0.01	−3.86	↓
592	Z-3-Me-pent-2-en-COOH-TMNO-naphth-1-yl	2.02	< 0.01	−5.12	↓

*ID* The unique number of the substance in this qualitative analysis, *VIP* variable importance in projection, *P-value* test probability with the sample data, *fold change* the arithmetic mean values of peak intensity, *Up* indicates that the content of metabolites increased, *Down* indicates that the content of metabolites decreases

**Fig. 3:**
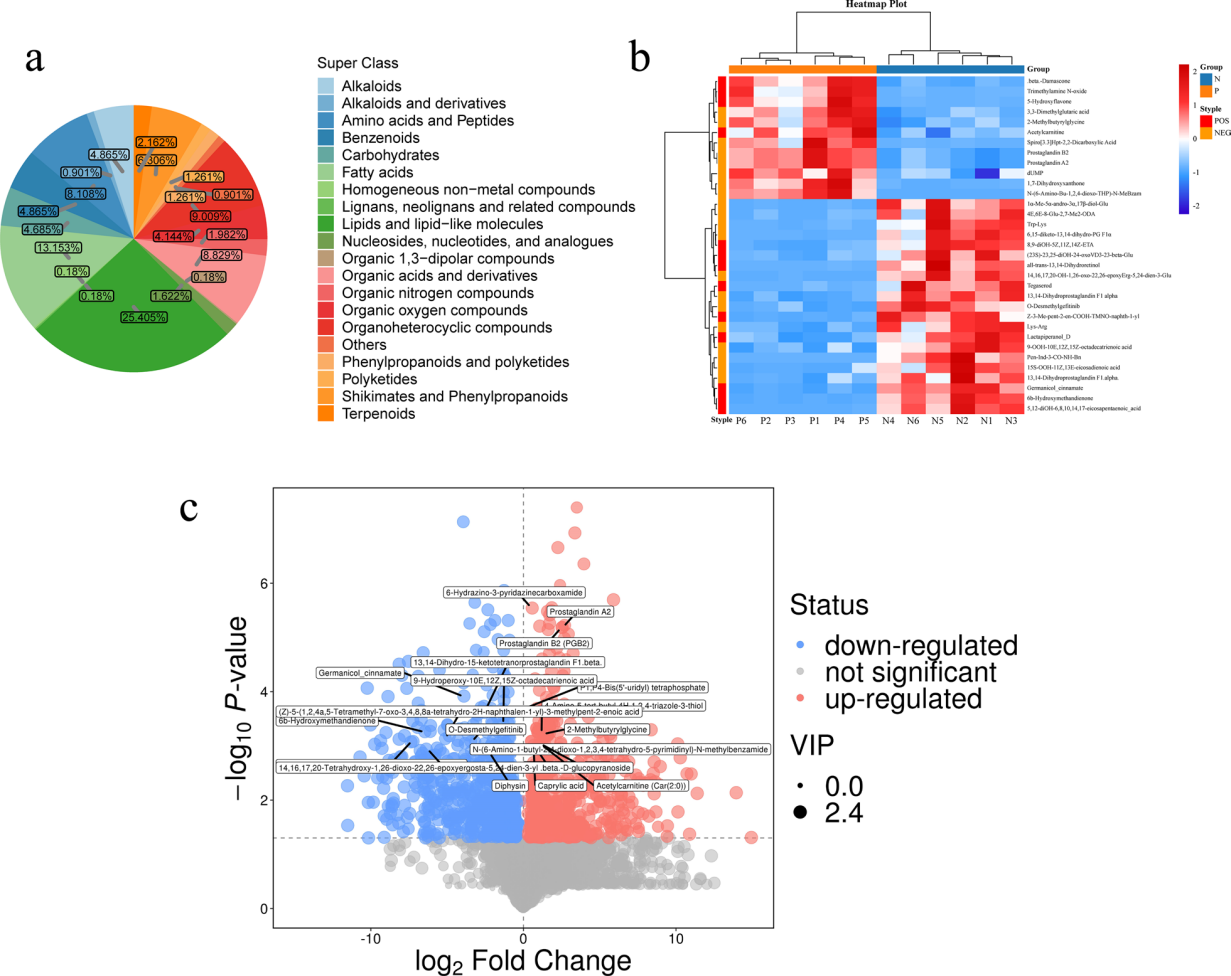
Multivariate statistical analysis of differential metabolites. **a** Donut plot of metabolite classification and proportions. Different color blocks represent different taxonomic categories, and percentages represent the percentage of metabolites belonging to that type in the total number of metabolites identified. **b** Heatmap of differentially abundant metabolites between the control and CE groups. The horizontal axis represents the names of the serum samples from the CE group and healthy control group, and the vertical axis represents the different metabolites. Red indicates that the substance is highly expressed in the group where it is located, blue indicates that the substance is low in the group where it is located, and the depth indicates the degree of influence. **c** Volcano map of differentially abundant metabolite screening results. Significantly upregulated metabolites are shown in red, significantly downregulated metabolites are shown in blue, and non-significantly differentiated metabolites are shown in gray

The metabolite classification and percentage ring results were 25.405%, 13.153%, 8.108%, 9.009%, 8.829%, and 6.306%, which mainly included lipids and lipid-like molecules, fatty acids, amino acids and peptides, organoheterocyclic compounds, organic acids and derivatives, and shikimates and phenylpropanoids (Fig. 3a). The hierarchical clustering of significantly different metabolite contents, metabolite heatmaps, and volcano plot data from the control group and CE group were clustered into one category each (Fig. 3b, c).

**Fig. 4:**
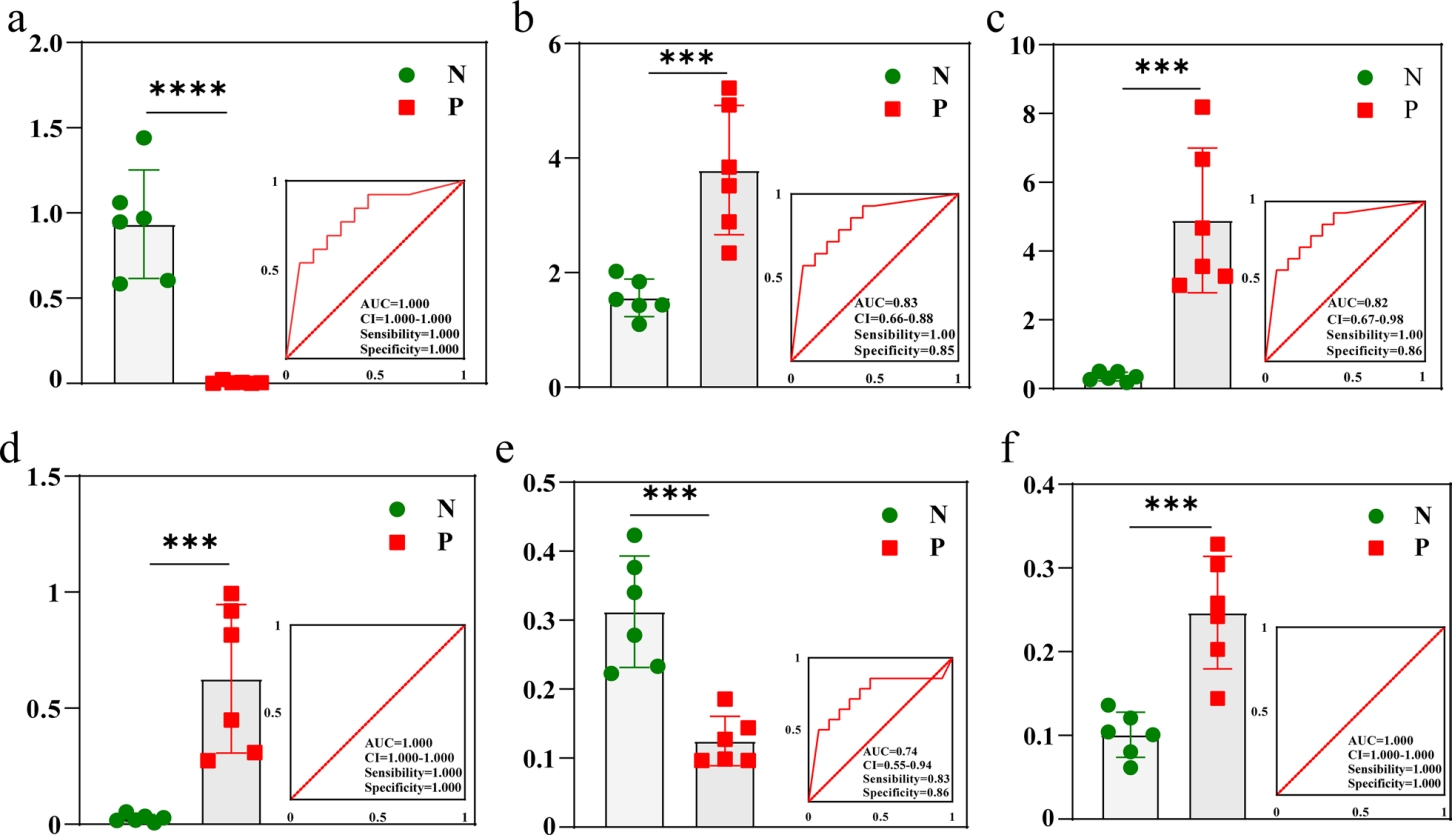
ROC analysis of potential biomarkers and determination of the predictive power of differentially abundant metabolites. **a** 2-Methylbutyrylglycine, **b** 3,3-dimethylglutaric acid, **c** 1,7-dihydroxyxanthone, **d** trimethylamine *N*-oxide, **e** 9-hydroperoxy-10E,12Z,15Z-octadecatrienoic acid, **f** 5,12-dihydroxy-6,8,10,14,17-eicosapentaenoic acid. AUC values closer to 1 indicate improved diagnostic effectiveness, while an AUC of 0.5 signifies complete ineffectiveness and lack of diagnostic value

Based on the 33 differentially abundant metabolites screened above, the diagnostic performance of the candidate markers was evaluated using the AUC of the ROC curve, and among the differentially abundant metabolites in the control and CE groups, one exhibited an AUC > 0.7, namely, 9-hydroperoxy-10E,12Z,15Z-octadecatrienoic acid. Additionally, five metabolites displayed an AUC > 0.8 including 1,7-dihydroxyxanthone, 2-methylbutyrylglycine, 3,3-dimethylglutaric acid, and 5,12-dihydroxy-6,8,10,14,17-eicosapentaenoic acid. Concerning trimethylamine *N*-oxide, six differentially abundant metabolites with good diagnostic efficacy for CE and high sensitivity (0.83–1.00) and specificity (0.86–1.00) were identified as potential serum biomarkers. These metabolites can be potentially used as more efficient serum diagnostic markers for CE, with clinical application (Fig. 4).

### Enrichment analysis of metabolic pathways for differentially abundant metabolites

**Fig. 5:**
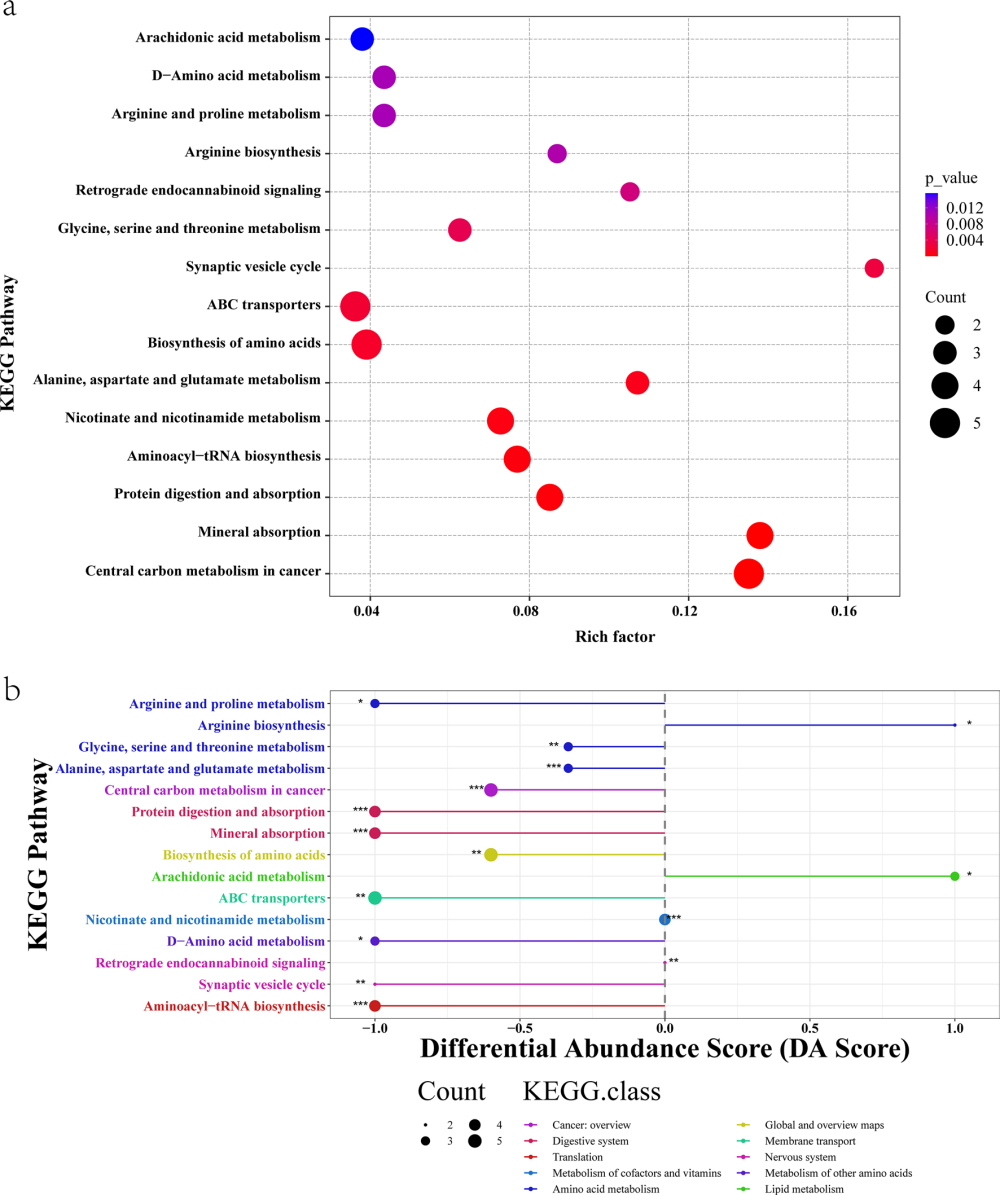
Bubble diagram of metabolic pathway enrichment of differentially abundant metabolites and differential abundance (DA) scores of metabolic pathways associated with different metabolites. **a** The vertical axis is the name of the metabolic pathway, and the horizontal axis is the enrichment factor. The greater the enrichment factor, the greater the enrichment degree. The larger the dot diameter, the greater the number of metabolites, and the *P-*value is the value of the hypergeometric test. **b** The horizontal coordinate represents the DA score, and the vertical coordinate represents the KEGG metabolic pathway name. The DA score reflects the overall change in all metabolites in the metabolic pathway; a score of 1 indicates a trend toward upregulation of the expression of all the annotated differentially abundant metabolites in the pathway, a score of −1 indicates a trend toward downregulation of the expression of all the annotated differentially abundant metabolites in the pathway, and the length of the line segment represents the absolute value of the DA score. The size of the dots indicates the number of annotated differentially abundant metabolites in the pathway, and larger dots indicate more differentially abundant metabolites in the pathway. The larger the dots, the greater the number of different metabolites in the pathway. The longer the distribution of the dots on the right side of the center axis, the more upregulated the overall expression of the pathway; the longer the distribution of the dots on the left side of the center axis, the more downregulated the overall expression of the pathway

The 33 differentially abundant metabolites were associated with a diverse range of biological processes, including energy metabolism, substance transport, and biosynthesis. Specifically, these pathways included amino acid metabolism, metabolism of cofactors and vitamins, metabolism of other amino acids, lipid metabolism, translation, digestion, the digestive system, membrane transport, and the nervous system. These metabolic pathways are significantly associated with the development of hydatid disease (Fig. 5a, b). The KEGG IDs of the 11 annotated metabolites are shown in Table 2. As the significant differences in metabolic pathways between the CE group and the control group increased, a progressively greater number of metabolic molecules became involved in specific pathways including fatty acid, hydroxyproline, creatine, glycine, l-phenylalanine, and l-leucine.

**Table 2:** Analysis of metabolic pathways via MetaboAnalyst based on all identified differentially abundant metabolites

Pathway	Total	Hits	Raw *P*	Impact	Hits
Arginine and proline metabolism	44	4	0.035	0.052	*N*-Acetylornithine; hydroxyproline; creatine; fumaric acid
Glycine, serine, and threonine metabolism	32	3	0.061	0.324	Dimethylglycine; glycine; creatine
Nicotinate and nicotinamide metabolism	13	2	0.051	0.256	Nicotinate d-ribonucleoside; niacinamide
Pyrimidine metabolism	37	2	0.288	0.01	Deoxycytidine; P1,P4-Bis(5′-uridyl) tetraphosphate
Alanine, aspartate, and glutamate metabolism	23	2	0.14	0.003	l-Asparagine; fumaric acid
Phenylalanine, tyrosine, and tryptophan biosynthesis	4	1	0.11	0.5	l-Phenylalanine
Phenylalanine metabolism	9	1	0.231	0.407	l-Phenylalanine
Valine, leucine, and isoleucine biosynthesis	11	1	0.275	0.333	l-Leucine
Primary bile acid biosynthesis	46	1	0.745	0.03	Glycine
Citrate (TCA) cycle	20	1	0.444	0.027	Fumaric acid
Glutathione metabolism	26	1	0.535	0.006	Glycine

Hits = the number of individuals within the current metabolic set that contribute to the pathway. Total = the total count of metabolites within the pathway. Raw *P* = the *P*-value derived from metabolic pathway enrichment analysis. Impact = the pathway’s importance score, indicating its relevance

### KEGG pathway annotation and regulatory network analysis of differentially abundant metabolites

**Fig. 6:**
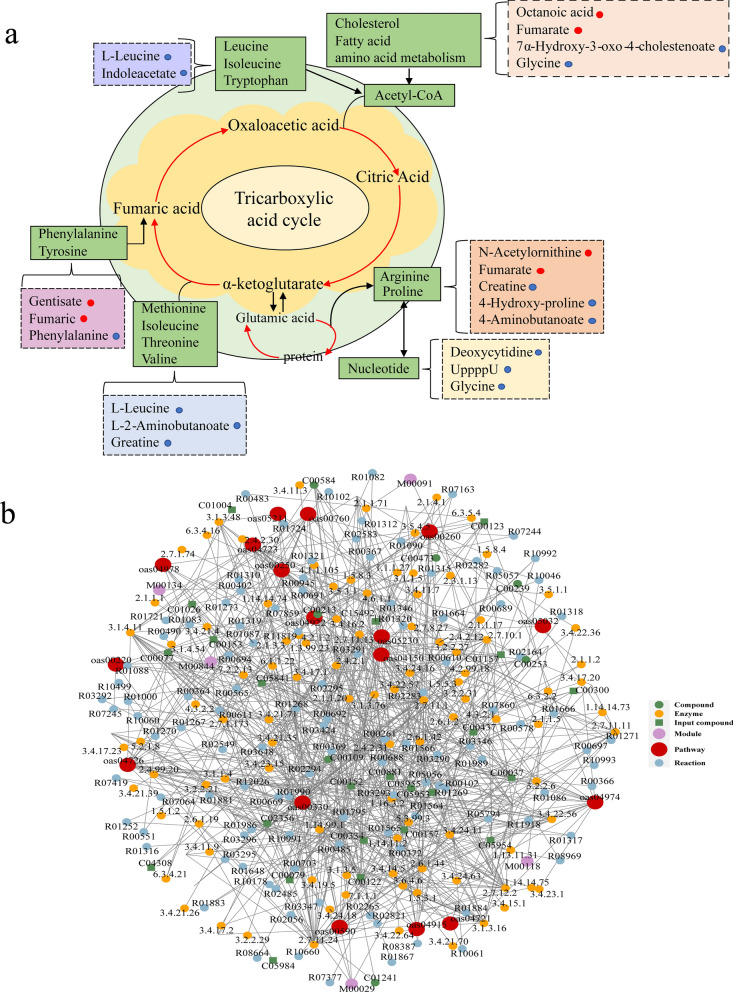
Correlation analysis of differentially abundant metabolites and network analysis for the CE and control groups. **a** Visual representation of metabolite abundance utilizes a color-coded system, where metabolites are depicted as dots. Bright red dots signify significantly upregulated metabolites, while bright blue dots indicate significantly downregulated metabolites. Additionally, metabolic pathways are outlined by green boxes, and directional flow of metabolic reactions is illustrated by connecting lines. **b** Red dots represent a metabolic pathway, yellow dots represent information on a substance-associated regulatory enzyme, green dots illustrate a background substance for a metabolic pathway, purple dots represent information on the molecular modules of a class of substances, blue dots show chemical interactions of a substance, and green squares represent differential substances obtained from this comparison

KEGG annotation analysis annotates only the pathways in which the differentially abundant metabolites are involved and provides a detailed examination of the interactions between these metabolites and the pathways in which they participate, as well as their effects. Metabolic networks can reflect the intersections between metabolic pathways as well as the targeting of potential enzymes and metabolites under specific study conditions, indicating how perturbations are propagated at the pathway level and how pathways interact with each other, thus enhancing the interpretability of the results. After obtaining matching information for each set of contrasting differentially abundant metabolites, we performed pathway searches and regulatory interaction network analysis on the KEGG database of the corresponding species *Ovis aries* (sheep) [37]. The results showed that the differentially abundant metabolites involved in the tricarboxylic acid cycle serve to satisfy the energy supply of the worm. Overall, the expression of genes related to regulatory effects was downregulated. Compared with those in the control group, only fenugreek was significantly upregulated in the CE serum samples, while glycine, creatine, phenylalanine, and leucine were significantly downregulated. The metabolites and metabolic pathways implicated in this complex metabolic network diagram revealed their involvement in cysticercosis development (Fig. 6a, b).

## Discussion

Surgical resection and drug therapy are the main treatment modalities for CE [38], and the early diagnosis of CE allows patients to be treated promptly, improving prognosis and survival. Compared with the imaging methods used for human CE, there are few diagnostic techniques available for studying CE in domestic animals infected as intermediate hosts.

The liver serves as a critical site for synthesis, metabolism, and mounting immune responses. Notably, it is the liver that harbors 70% of cysts in sheep infected with cysticercosis and patients suffering from cerebral cysticercosis, making it a vital location for diagnosis and treatment. In previous studies, CE mouse models of secondary infection established by the intraperitoneal injection of the protoscolex were used to screen potential diagnostic biomarkers. However, the present study aimed to identify serum biomarkers for CE in sheep through untargeted metabolomic analysis using LC–MS/MS technology. We identified numerous specific metabolites in the serum of the sheep contaminated with CE including 1,7-dihydroxyxanthone, 2-methylbutyrylglycine, 3,3-dimethylglutaric acid, 5,12-dihydroxy-6,8,10,14,17-eicosapentaenoic acid, 9-hydroperoxy-10E,12Z,15Z-octadecatrienoic acid, and trimethylamine *N*-oxide 6, providing numerous diagnostic biomarkers in clinical practice.

The genomic and transcriptomic sequencing of *E. granulosus* showed that despite having a complete glycolysis, tricarboxylic acid cycle, and pentose phosphate pathway, this parasitic worm cannot synthesize amino acids, nucleotides, or lipids. This suggests that *E. granulosus* relies on the host’s metabolic resources for its growth and development [39, 40]. Parasite–host interactions have always been a hot topic in parasitology research, and the use of cutting-edge omics technologies like post-genomic and proteomic diagnostic techniques has increased in recent years. The study of the metabolic behavior of the parasite itself is important because it can elucidate the metabolic relationship between the host and parasite, and an understanding of the metabolic profile of the parasite can also facilitate the development of improved parasite treatment methods, such as killing the parasite by affecting its nutrient uptake. Obtaining energy from the host is vital for the growth and development of the parasite, and blocking the energy acquisition of the parasite is an effective means of inhibiting its growth. Compared with those in the control group, the serum levels of glycine, creatine, leucine, phenylalanine, and other major metabolites were lower in the CE group, possibly because *E. granulosus* cannot synthesize cholesterol by itself, and must obtain cholesterol from the host, participating in lipid and amino acid metabolism pathways through the action of enzymes [39].

Common analytical methods for metabolomics include chromatography–capillary electrophoresis, proton nuclear magnetic resonance, MS, HPLC, and gas chromatography. Here, LC–MS non-targeted metabolism techniques are widely used to analyze differential metabolites in the serum of patients with hepatocellular carcinoma, cirrhosis, and viral hepatitis to screen for potential serum biomarkers. In the field of parasitology, LC–MS non-targeted metabolism techniques are some of the most frequently used metabolomics methods, especially for the detection of metabolites in serum samples [41]. In 1992–1993, Novak et al. [42, 43] used metabolomics to study alterations in the organ metabolism of *E. multilocularis*-infected meristematic tissues and found similarly low glycine levels in the livers and spleens infected with *E. multilocularis*. Garg et al.’s [44] study is a great example of how metabolomics can be used to differentiate between parasites, specifically *E. granulosus* and cysticercus. By comparing the metabolic patterns of the two parasites, they were able to identify specific metabolites that could be used as biomarkers for differentiation. Also, particularly high levels of fenugreek were observed when the cysts of *E. granulosus* were active, but not in cysticercus cysts. Based on these results, in 2002, Garg et al. [45] made a taxonomic correlation with the metabolomes of a small number of viable and non-viable cyst samples using the metabolomics of cyst fluid from humans and sheep. Building upon their research, they successfully distinguished between fertile and sterile cysts. The study also revealed the presence of substances such as fenugreek in the cyst fluid of fertile cysts, which was corroborated by histopathological observations of the fertile cyst walls. Hosch et al. [12] built upon earlier findings and validated the presence of fenugreek in *Echinococcus* cysts, and by analyzing a larger cohort of samples, primarily sourced from liver infections, they demonstrated that fertile cysts consistently exhibited higher levels of fenugreek than infertile cysts, using metabolomics technology, which is consistent with the findings of the present study. It is therefore hypothesized that fenugreek is not present in sterile cysts and that fenugreek can be used to determine the fertility status of *E. granulosus*, which is the most important factor in the diagnosis of encapsulated cysts. In 2019, Ritler et al. [46] used metabolomics to characterize the metabolites consumed and released by *E. multilocularis* tapeworms under anaerobic conditions and showed that glucose was the most consumed metabolite by *E. multilocularis* tapeworms. Thus, the therapeutic significance of glycolysis inhibitors in the treatment of encapsulated parasites can be fully considered in the fight against encapsulated parasites. Interestingly, albendazole, a broad-spectrum anthelmintic drug with mature clinical applications, can inhibit glucose uptake by the parasite, leading to glycogen depletion in the worm. According to a metabolomics study, it is also capable of inhibiting the fenugreek acid reductase system, leading to the disruption of ATP production, thereby preventing the survival and reproduction of the worm [47].

In the current study, we analyzed the significant changes in metabolites in the serum of *E. granulosus*-infected sheep using LC–MS/MS technology combined with multivariate statistical methods. The results showed that the changes were closely related to amino acid metabolism and fatty acid metabolism pathways and that the energy metabolism of the host may interfere with the process of infection. These metabolic differences may provide a reference for understanding the biological mechanisms and diagnostic biomarkers involved in the process of early CE infection. In addition, the significant changes in the serum levels of glycine, arginine, l-isoleucine, and l-valine in sheep in the CE group may be attributed to liver damage caused by CE lesions parasitizing the liver, resulting in disruption of the metabolic balance of amino acids in the body. This led to the conclusion that the metabolites important for CE were valine, leucine, isoleucine, glycine, and fenugreek. Further research is warranted to elucidate the molecular mechanisms responsible for the effect of lipid and amino acid metabolites on the pathogenicity of *Echinococcus*. Through metabolomics, we can understand the characteristics of *E. granulosus* growth and metabolic development in the host, screen specific metabolic targets and develop relevant drugs for these organisms, and provide novel ideas for the treatment of the encapsulated disease. The small sample size of the current study is a potential limitation, and the results should be validated with a larger sample and more diverse host groups. Also, additional possible steps for CE lesions in the host should be considered in future experiments.

## Data Availability

Data are provided within the manuscript.

## Abbreviations

KEGG: Kyoto Encyclopedia of Genes and Genomes
OPLS-DA: Orthogonal projections to latent structures-discriminant analysis
IDA: Information-dependent acquisition
CE: Cystic echinococcosis
QC: Quality control
LC–MS/MS: Liquid chromatography–tandem mass spectrometry
ESI: Electrospray ionization
PCA: Principal component analysis
VIP: Variable importance in projection
ROC: Receiver operating characteristic
AUC: Area under the curve
